# 20-Hydroxyeicosatetraenoic acid antagonist attenuates the development of malignant hypertension and reverses it once established: a study in Cyp1a1-Ren-2 transgenic rats

**DOI:** 10.1042/BSR20171496

**Published:** 2018-09-12

**Authors:** Lenka Sedláková, Soňa Kikerlová, Zuzana Husková, Lenka Červenková, Věra Čertíková Chábová, Josef Zicha, John R. Falck, John D. Imig, Elzbieta Kompanowska-Jezierska, Janusz Sadowski, Vojtěch Krátký, Luděk Červenka, Libor Kopkan

**Affiliations:** 1 Center for Experimental Medicine, Institute for Clinical and Experimental Medicine, Prague, Czech Republic; 2 Department of Nephrology, 1st Faculty of Medicine, Charles University, Prague, Czech Republic; 3 Institute of of Physiology, Czech Academy of Sciences, Prague, Czech Republic; 4 Department of Biochemistry, University of Texas Southwestern Medical Center, Dallas, TX, U.S.A.; 5 Department of Pharmacology and Toxicology, Medical College of Wisconsin, WI, U.S.A.; 6 Department of Renal and Body Fluid Physiology, M. Mossakowski Medical Research Centre, Polish Academy of Science, Warsaw, Poland; 7 Department of Pathophysiology, 2nd Faculty of Medicine, Charles University, Prague, Czech Republic

**Keywords:** cytochrome p450 metabolites, malignant hypertension, renin-angiotensin system, 20-hydroxyeicosatetraenoic acid

## Abstract

We hypothesized that vascular actions of 20-hydroxyeicosatetraenoic acid (20-HETE), the product of cytochrome P450 (CYP450)-dependent ω-hydroxylase, potentiate prohypertensive actions of angiotensin II (ANG II) in Cyp1a1-Ren-2 transgenic rats, a model of ANG II-dependent malignant hypertension. Therefore, we evaluated the antihypertensive effectiveness of 20-HETE receptor antagonist (AAA) in this model. Malignant hypertension was induced in Cyp1a1-Ren-2 transgenic rats by activation of the *renin* gene using indole-3-carbinol (I3C), a natural xenobiotic. Treatment with AAA was started either simultaneously with induction of hypertension or 10 days later, during established hypertension. Systolic blood pressure (SBP) was monitored by radiotelemetry, indices of renal and cardiac injury, and kidney ANG II levels were determined. In I3C-induced hypertensive rats, early AAA treatment reduced SBP elevation (to 161 ± 3 compared with 199 ± 3 mmHg in untreated I3C-induced rats), reduced albuminuria, glomerulosclerosis index, and cardiac hypertrophy (*P*<0.05 in all cases). Untreated I3C-induced rats showed augmented kidney ANG II (405 ± 14 compared with 52 ± 3 fmol/g in non-induced rats, *P*<0.05) which was markedly lowered by AAA treatment (72 ± 6 fmol/g). Remarkably, in TGR with established hypertension, AAA also decreased SBP (from 187 ± 4 to 158 ± 4 mmHg, *P*<0.05) and exhibited organoprotective effects in addition to marked suppression of kidney ANG II levels. In conclusion, 20-HETE antagonist attenuated the development and largely reversed the established ANG II-dependent malignant hypertension, likely via suppression of intrarenal ANG II levels. This suggests that intrarenal ANG II activation by 20-HETE is important in the pathophysiology of this hypertension form.

## Introduction

Hypertension is not only the major independent risk factor of myocardial infarction, stroke, and progression of chronic kidney disease, but also the most important single contributor to the global disease burden and to global mortality [1,2]. Malignant hypertension is the most severe and, if untreated, a fatal form of this disease. Its hallmark is acute extreme elevation of blood pressure (BP), which is associated with severe end-organ damage, especially the kidney [3–7]. Before introduction of modern antihypertensive drugs, the prognosis was gloomy with high mortality rates [3–5]. Even though malignant hypertension is a rare condition affecting 1–2% of all hypertensive patients, its incidence and prevalence do not decrease over time [4–6] and despite the improvement in antihypertensive treatment regimes it remains a life-threatening condition and a serious economic burden on public health services [6–8]. Thus, there is a great need for new therapies to combat severe hypertension and the associated organ damage. However, the prerequisite for the development of new pharmacological measures is a more detailed understanding of the pathophysiology of malignant hypertension, which is still to come.

It will be recalled that seminal studies from Laragh group revealed that abnormal activation of the renin–angiotensin system (RAS) plays a crucial role in the pathophysiology of malignant hypertension [9,10] and the notion that inappropriate activation of the RAS importantly contributes to the transition to the malignant phase was later confirmed by many other studies [11–14]. Nevertheless, the exact nature of the alterations in the RAS activity in the pathophysiology of malignant hypertension is still incompletely understood. It is currently believed that disturbed interaction of RAS with other vasoactive systems (rather than simply RAS activation) might be here important [6,10–12,15].

Within this concept, particular attention was recently focussed on 20-hydroxyeicosatetraenoic acid (20-HETE), the product of the ω-hydroxylation of arachidonic acid by cytochrome P450 (CYP450), because an increasing body of evidence suggests that 20-HETE’s vascular actions explain its prohypertensive properties [16–20]. In addition, it has been documented that functional interaction between RAS and 20-HETE at the vascular level is complex and both components work in concert to promote the development of hypertension [21–26]. Moreover, recent studies suggested that 20-HETE might represent an upstream effector molecule for the activation of RAS in the vasculature [23,27–29].

Given the aforementioned evidence, in the present study we tested the hypothesis that increased vascular actions of 20-HETE contribute to the development of angiotensin II (ANG II)-dependent malignant form of hypertension. We employed here inbred transgenic rats with inducible hypertension (strain name: Cyp1a1-Ren-2); these animals express the mouse *Ren-2* renin gene under control of the Cyp1a1 promoter. After dietary administration of indole-3-carbinol (I3C), the expression of the Cyp1a1 promoter is rapidly enhanced, with marked activation of *Ren-2* renin gene in the liver, with subsequent increase in ANG II generation and development of ANG II-dependent malignant hypertension [30]. We and others have demonstrated that the level of BP can be precisely controlled in a dose- and time-dependent manner [31–36]; evidently, the model greatly facilitates exploration of the role of the RAS in the pathophysiology of malignant hypertension.

In the present study, we employed this model to examine early chronic oral administration of a new 20-HETE receptor antagonist effect on the development of ANG II-dependent malignant hypertension. To make the study even more relevant to the situation of patients already in the malignant phase of hypertension, we also examined if the treatment would attenuate hypertension and end-organ damage in our Cyp1a1-Ren-2 transgenic rats at the established malignant phase.

Furthermore, to gain more insight into the possible intrarenal interactions for the whole spectrum of CYP450-derived metabolites and the RAS, we determined the effects of 20-HETE antagonist treatment on intrarenal concentrations of 20-HETE and also of other products of CYP450 metabolites [37], as well as on intrarenal ANG II. Finally, we also examined the effects of ANG II type 1 (AT_1_) receptor inhibition on the aforementioned parameters and assessed renal hemodynamics and vascular responsiveness to vasoactive agents in this model.

## Methods

### Ethical approval, animals, diets, and chemicals

The studies were approved by the *Animal Care and Use Committee of the Institute for Clinical and Experimental Medicine (IKEM)*, Prague, and of the 2nd Faculty of Medicine, Charles University, Prague, which are in accord with the *European Convention on Animal Protection and Guidelines on Research Animal Use.* An inbred transgenic rat with inducible hypertension, Cyp1a1-Ren-2, was used as a model of malignant hypertension. At IKEM facility accredited by the Czech Association for Accreditation of Laboratory Animal Care, the rats were bred from the stock animals supplied by the Center for Cardiovascular Science, University of Edinburgh, U.K. (we acknowledge the generous gift of Professor Mullins). Rats had free access to tap water and were fed either a rat chow without I3C (non-induced groups) or one containing 0.3% I3C (I3C-induced groups). Previous studies have clearly demonstrated that chronic dietary administration of I3C at this dose induces malignant hypertension with marked activation of the endogenous RAS. In addition, this is accompanied by a rapid increase in BP, body weight (BW) loss, pressure diuresis and natriuresis, and severe renal vasoconstriction and ischemia, which all are typical signs of ANG II-dependent malignant hypertension [12–14,30–36,38–40]. Since during the generation of this model the transgene was integrated into the Y chromosome, only male rats were used [30]. Otherwise the chow (SEMED, Prague, Czech Republic) contained salt and protein at normal concentrations (0.45% NaCl, 19–21% protein). The animals were kept on a 12-h/12-h light/dark cycle.

**Figure 1:**
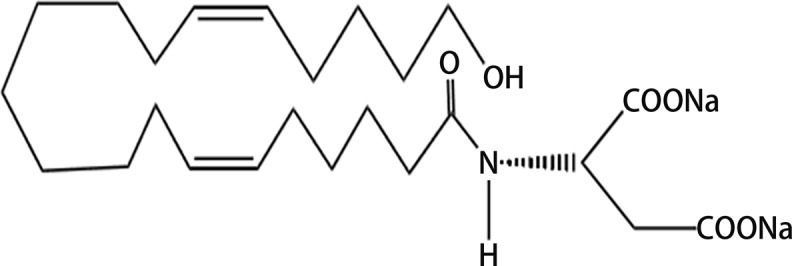
Chemical structure of 20-HETE antagonist – (N-disodium succinate-20-hydroxyeicosa-6(Z),15(Z)-diencarboxamide) (chemical formula: C_25_H_43_NNa_2_O_6_; MW: 499.59)

We employed the water-soluble 20-HETE receptor antagonist (name: AAA), chemical structure: (N-disodium succinate-20-hydroxyeicosa-6(Z),15(Z)-diencarboxamide) (Figure 1), that was given in drinking water at the concentration adjusted to yield a daily dose of 10 mg.kg^−1^.day^−1^, based on a recent study [41]. AAA with its low molecular weight (MW = 499.59) displays good solubility and stability in stability in water so that there is no need of additional dissolving agent. These properties ensure its better efficacy and longer half-life. In addition, our preliminary studies have shown that plasma AAA concentrations in treated non-induced as well as I3C-induced Cyp1a1-Ren-2 transgenic rats exceeded the IC_50_ level. AAA was designed and synthesized in J.R.F.’s laboratory. Losartan (Lozap, 100 mg/l in drinking water, Zentiva, Prague, Czech Republic) was used as AT_1_ receptor blocker, because we and others previously showed that this dose completely blocked the development of hypertension in Cyp1a1-Ren-2 transgenic rats after induction of the *renin* gene [36,41].

### Experimental design

#### Series 1: Effects of treatments with 20-HETE receptor antagonist or AT_1_ receptor blocker starting together with induction of renin gene expression (‘early treatment’ protocol) on BP, albuminuria, cardiac hypertrophy, renal morphology, and kidney concentrations of 20-HETE, epoxyeicosatrienoic acids, and dihydroxyeicosatrienoic acids

In accordance with the recommendation for BP measurement in experimental animals, we employed a radiotelemetry system [42]. Rats were anesthetized with a combination of tiletamine, zolazepam (Zoletil, Virbac SA, Carros Cedex, France; 8 mg.kg^−1^), and xylazine (Rometar, Spofa, Czech Republic; 4 mg.kg^−1^) intramuscularly, and TA11PA-C40 radiotelemetric probes (Data Sciences International, St. Paul, MN, U.S.A.) were implanted for direct BP measurements as described previously [34,39,40]. The rats were allowed 10 days to recover before basal BP was recorded and only animals with stable BP records at the end of this recovery period were used for experiments. Basal BP was determined for 3 days and then induction of *renin* gene was started and continued until the end of the experiment that lasted 12 days. The treatment with 20-HETE antagonist or AT_1_ receptor blocker was started simultaneously with initiation of dietary administration of I3C. In the animals implanted with radiotransmitters, 24-h urine collections were performed in metabolic cages prior (day 3) and during (days 3 and 10) the experiments by methods described previously [31,34,39,40,43]. At the end of experiments, all rats were killed with an overdose of thiopental sodium (Sandoz, Basel, Switzerland) and the kidneys were removed for assessment of renal injury. Renal glomerular damage and tubulointerstial injury were assessed by methods described in our previous studies, which allowed comparison of the present results with those of our previous studies of pathophysiology of hypertension-associated end-organ damage [31,44–46]. We used the ratio of left ventricle weight to tibia length to evaluate the degree of cardiac hypertrophy, since we and others have previously demonstrated that this is the most suitable index to assess cardiac hypertrophy under conditions of a significant loss of BW [31,33,34,45,47], which was the case here.

The following groups of Cyp1a1-Ren-2 transgenic rats were examined:

1. I3C-induced/untreated (*n*=9)
2. I3C-induced/20-HETE antagonist (*n*=9)
3. I3C-induced/AT_1_ receptor blocker (*n*=8)
4. Non-induced/untreated (*n*=7)
5. Non-induced/20-HETE antagonist (*n*=7)
6. Non-induced/ AT_1_ receptor blocker (*n*=7)

Separate groups of animals (*n*=9 in each) were exposed to the same experimental protocol as described above, and on day 12 the animals were killed by decapitation. The reason was that plasma and tissue ANG II concentrations in anesthetized animals are higher than those obtained from decapitated conscious rats, and that normotensive animals exhibit a greater increase in renin secretion in response to anesthesia and surgery than observed for ANG II-induced hypertensive intrarenal renin-depleted animals [32,34,48,49]. Whole-kidney ANG II concentrations were assessed by RIA as described in detail in our previous studies [32,33,39,48,49]. 20-HETE was measured in kidney cortex from samples that were extracted as described previously [50], the extracts were separated by reverse-phase HPLC and analyzed by negative-mode ESI and MS/MS as described previously [50]. The samples were frozen in liquid nitrogen and stored at −80°C till analysis. Alkaline hydrolysis was performed in homogenized tissue. The columns with samples were washed with methanol-water (1:1). The samples were eluted with hexane-ethyl acetate with acetic acid. The organic extracts were evaporated to dryness under reduced pressure and reconstituted in methanol for HPLC purification on an Agilent 1200SL system. In addition, kidney concentrations of epoxyeicosatrienoic acids (EETs) and their metabolites (dihydroxyeicosatrienoic acids (DHETEs)), specifically 5,6-EETs; 8,9-EETs; 11,12-EETs; and 14,15-EETs were measured separately and then pooled for final presentation of the EETs/DHETEs ratio as described previously [40,45,50]. The results were corrected to gram of protein.

#### Series 2: Effects of treatments with 20-HETE receptor antagonist or AT_1_ receptor blocker in Cyp1a1-Ren-2 transgenic rats with established hypertension (‘late treatment’ protocol) on BP, albuminuria, cardiac hypertrophy, and kidney ANG II levels

The protocol as described for series 1 was used except that the treatment with 20-HETE antagonist or AT_1_ receptor blocker was initiated 10 days after induction with I3C, in the phase of established malignant hypertension, and lasted 12 days. In addition, plasma angiotensin-converting enzyme (ACE) activity was measured by an enzymatic assay employing the commercially available kit (ACE kinetic, Bühlmann Laboratories AG, Schönenbuch, Switzerland). Since EDTA inhibits ACE activity, serum samples (collected without anticoagulant) were used here. The results are expressed in ACE activity units; one unit is defined as the amount of enzyme required to release one μmol of hippuric acid per minute and per liter of serum at 37°C.

In this series, the following four groups of Cyp1a1-Ren-2 transgenic rats were examined:

1. I3C-induced/untreated (*n*=8)
2. I3C-induced/20-HETE antagonist (*n*=8)
3. I3C-induced/AT_1_ receptor blocker (*n*=8)
4. Non-induced/untreated (*n*=6)

#### Series 3: Effects of treatments with 20-HETE receptor antagonist or AT_1_ receptor blocker on renal hemodynamics and vascular responsiveness to vasoactive agents in Cyp1a1-Ren-2 transgenic rats with established hypertension (‘late treatment’ protocol)

In this series, the same four groups of Cyp1a1-Ren-2 transgenic rats were examined as in the series 2 (*n*=8–9 in each group). After 3 days of treatment, the animals were anesthetized with thiopental sodium (Sandoz, Basel, Switzerland; 50 mg.kg^−1^) and placed on a thermoregulated table to maintain body temperature at 37–37.5°C. A tracheostomy was performed to maintain a patent airway, and the exterior end of the tracheal cannula was placed inside a small plastic chamber, into which humidified 95% O_2_–5% CO_2_ mixture was continuously passed to improve the stability of arterial pressure in anesthetized rats, as described previously [43]. The right jugular vein was catheterized with PE-50 tubing for intravenous administration of solutions, additional anesthetic as required and drugs. The right femoral artery was cannulated to allow continuous monitoring of arterial BP via a pressure transducer and data-acquisition system (model MLT 1050; PowerLab/4SP; ADInstruments, Chalgrove, U.K.) and blood sampling. The left kidney was exposed via a flank incision, isolated from the surrounding tissue and placed in a lucite cup, and the ureter was cannulated with a PE-10 catheter. Finally, an ultrasonic transient time flow probe (1RB, Transonic Systems, Altron Medical Electronic GmbH, Germany) connected to a Transonic flowmeter was placed around the left renal artery and renal blood flow (RBF) was recorded using a computerized acquisition system. During surgery, an isotonic saline solution containing BSA (6%) (Sigma Chemical Co., Prague, Czech Republic) was infused at a rate of 20 μl.min^−1^. The rats were allowed to recover for 45 min after completion of surgery and then isotonic saline solution containing polyfructosan (Inutest, Laevosan, Linz/Donau, Austria) (7.5%) was infused at the same volume infusion rate. The experimental protocol consisted of two 30-min urine collections to determine renal hemodynamic and excretory parameters in these rats as described previously [43,44]. After urine collection, intravenous boluses were administered to assess the vascular responsiveness to ANG II (10 and 20 ng), acetylcholine (ACh, 50 and 100 ng) in 5-min intervals. At the end of experiment, continuous i.v. infusion of nitric oxide synthase inhibitor (l-NAME) (50 μg.min^−1^.kg^−1^) was given till maximal reduction in RBF.

Urine volume was measured gravimetrically. Urinary sodium concentration was determined by flame photometry. Polyfructosan in plasma and urine was measured colorimetrically and polyfructosan clearance was used as an estimate of glomerular filtration rate (GFR). The values were calculated per gram of kidney weight. Renal vascular resistance (RVR) was calculated by dividing the mean arterial pressure by RBF.

### Statistical analyses

All values are expressed as mean ± S.E.M. Using the GraphPad Prism software (Graph Pad Software, San Diego, CA, U.S.A.), analysis was done by one-way ANOVA when appropriate (e.g. for the analysis of ANG II levels). ANOVA for repeated measurements, followed by Student–Newman–Keuls test was done for within-group analysis (e.g. for the analysis of systolic BP (SBP)**.** Values exceeding the 95% probability limits (*P*<0.05) were considered statistically significant.

## Results

### Series 1: ‘Early treatment’ protocol

**Figure 2:**
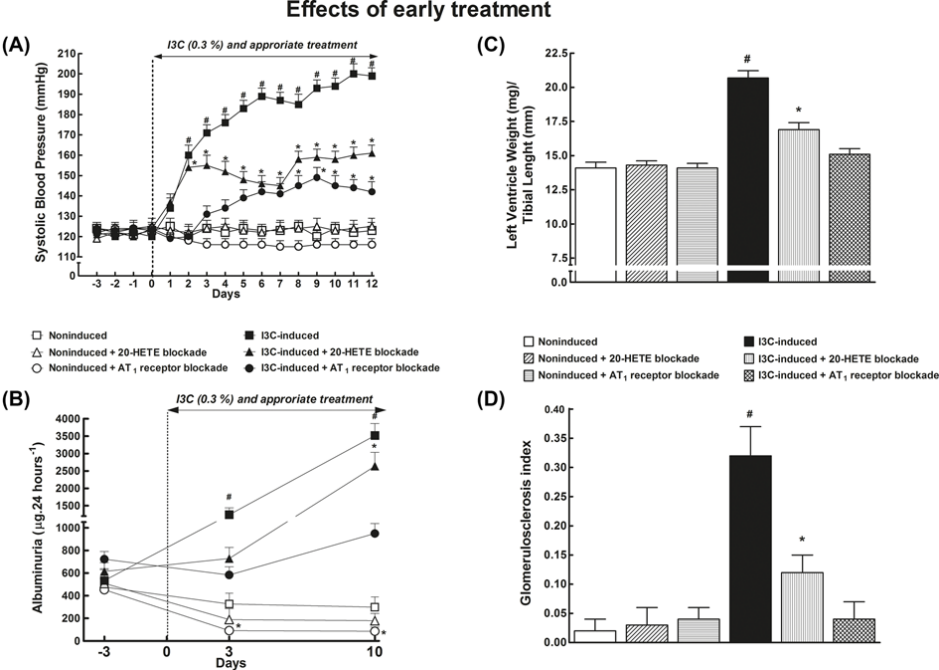
Comparison of 20-HETE receptor antagonist and ARB - early treatment Time course of SBP (**A**), albuminuria (**B**), the data on left ventricle weights normalized by tibial length (**C**), and glomerulosclerosis index (**D**) in I3C-induced and non-induced Cyp1a1-Ren-2 transgenic rats, and effects of 20-HETE antagonist or AT_1_ receptor antagonist treatment; *n*=6–8 per group. *P<0.05 compared with basal values (or compared with non-induced rats in the case of left ventricle weights and glomerulosclerosis index). ^#^*P*<0.05 compared with * marked values at the same time point (or compared with all the other groups of rats in the case of left ventricle weights and glomerulosclerosis index).

As shown in Figure 2A, SBP in non-induced rats remained within the normotensive range throughout the experiment and was not altered by 20-HETE or AT_1_ receptor blockade. I3C-induction resulted in severe hypertension (final SBP: 199 ± 3 mmHg on day 12 of induction). 20-HETE receptor antagonist significantly attenuated the development of hypertension across the whole treatment time (SBP was reduced by approximately 30 mmHg). AT_1_ receptor blockade was even more effective: on the final day 12 of induction SBP was only 142 ± 3 mmHg. In each group of non-induced rats, a slight progressing gain in BW was seen, ultimately by approximately 20 g, whereas in untreated I3C-induced rats the development of hypertension was accompanied by a profound BW loss (by approximately 70 g). Moreover, these rats displayed hunched posture, piloerection, and polydipsia, the typical features accompanying malignant hypertension [13–19]; notably, these phenotype characteristics were absent from I3C-induced rats treated with 20-HETE antagonist or AT_1_ receptor antagonist.

Figure 2B shows that untreated non-induced rats showed only minimal proteinuria throughout the experiment which was not affected by 20-HETE receptor antagonist treatment whereas AT_1_ receptor blockade lowered albuminuria as compared with untreated non-induced rats. Untreated I3C-induced rats displayed pronounced albuminuria: approximately seven-fold increase on day 10 of induction was seen, compared with preinduction values. This increase was attenuated in rats treated with 20-HETE antagonist and prevented by treatment with AT_1_ receptor antagonist.

Untreated I3C-induced rats showed marked cardiac hypertrophy as compared with non-induced rats (Figure 2C). 20-HETE receptor antagonist attenuated the hypertrophy, whereas AT_1_ receptor blockade abolished it.

**Figure 3:**
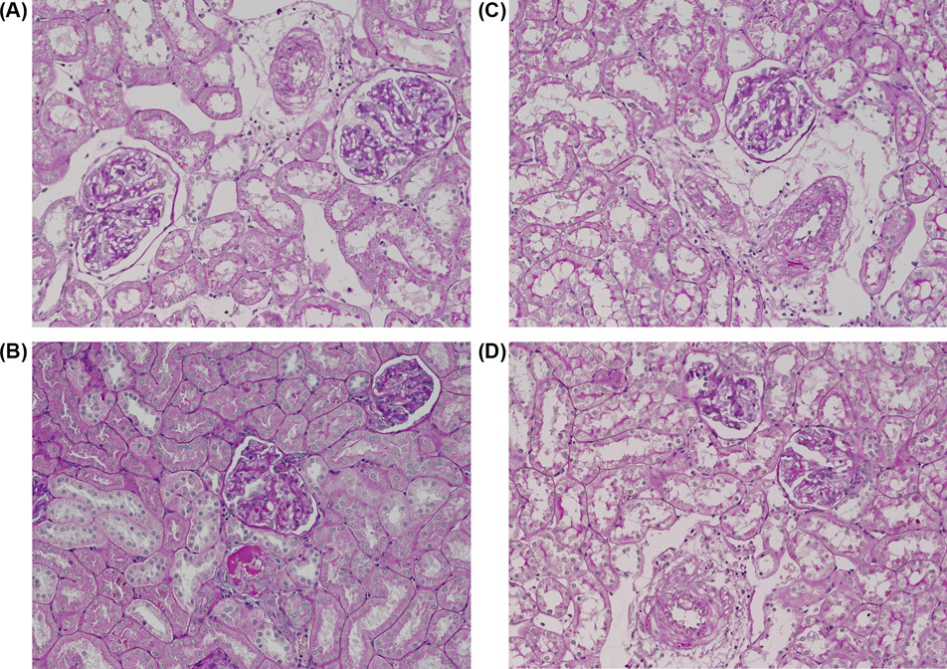
Representative histological images used for calculation of the glomerulosclerosis index (**A**) Untreated non-induced Cyp1a1-Ren-2 transgenic rats – normal renal cortex, (**B**) untreated I3C-induced Cyp1a1-Ren-2 transgenic rats – interlobular artery with fibrinoid necrosis, glomerulus (in the center) with insudation, (**C**) I3C-induced Cyp1a1-Ren-2 transgenic rats treated with 20-HETE antagonist – interlobular artery with segmental fibrinoid necrosis, (**D**) I3C-induced Cyp1a1-Ren-2 transgenic rats treated with AT_1_ receptor antagonist – interlobular artery with small segmental fibrinoid necrosis. Dashed lines encircle the large vessels. Magnification 200×. Periodic acid–Schiff staining.

As shown in Figure 2D, untreated non-induced rats displayed minimal glomerulosclerosis that was not altered by 20-HETE antagonist or AT_1_ receptor antagonist. In contrast, the glomerulosclerosis index was significantly increased in untreated I3C-induced rats; this increase being attenuated by 20-HETE receptor antagonist treatment and abolished by AT_1_ receptor blockade. Representative histology images that were used for calculation of the glomerulosclerosis index are shown in Figure 3.

**Figure 4:**
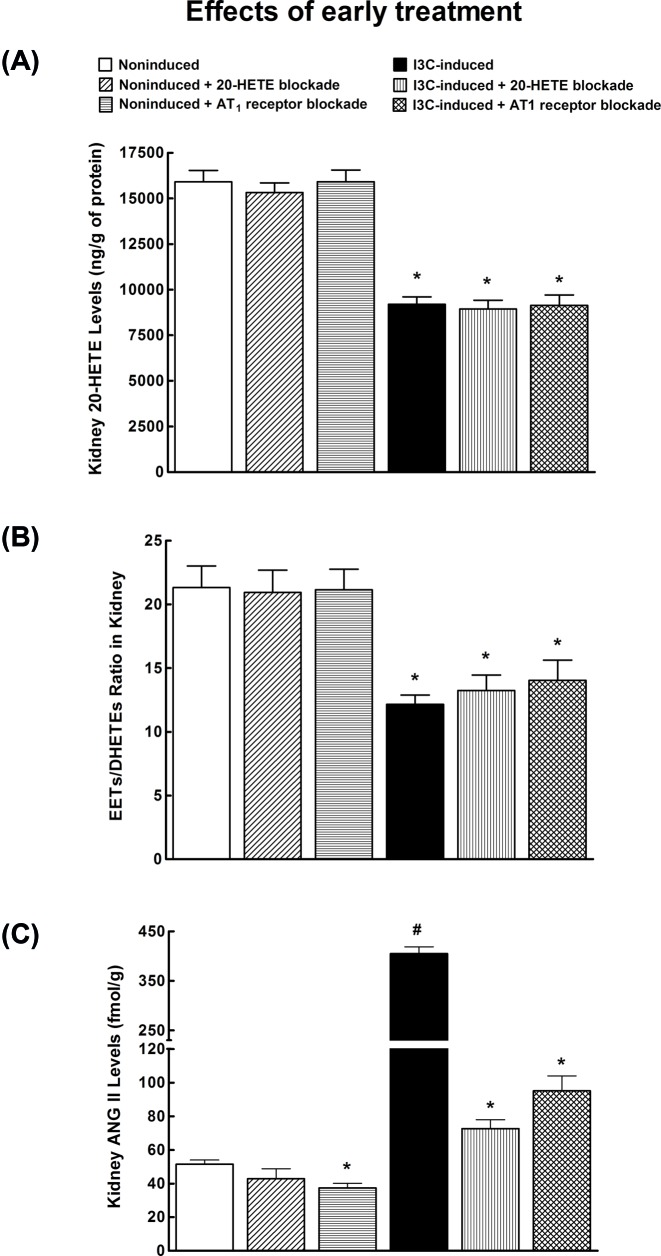
Comparison of 20-HETE receptor antagonist and ARB - early treatment (**A**) Kidney 20-HETE concentration, (**B**) kidney EETs to DHETEs ratio, and (**C**) kidney ANG II levels in I3C-induced and non-induced Cyp1a1-Ren-2 transgenic rats, and effects of 20-HETE antagonist and AT_1_ receptor antagonist treatment. *n*=6–8 per group. *P<0.05 compared with untreated non-induced rats. ^#^*P*<0.05 compared with all other values.

Figure 4A shows that untreated I3C-induced rats exhibited significantly lower renal concentration of 20-HETE as compared with untreated non-induced rats. The treatment with 20-HETE antagonist or AT_1_ receptor antagonist did not alter renal concentrations of 20-HETE, similarly in non-induced and I3C-induced rats.

Figure 4B shows the intrarenal availability of biologically active epoxy fatty acids expressed as the ratio of biologically active EETs to the almost inactive DHETEs. This ratio was significantly lower in I3C-induced rats as compared with non-induced rats. 20-HETE antagonist and AT_1_ receptor antagonist did not alter it, similarly in non-induced and I3C-induced rats.

Untreated I3C-induced rats exhibited eight-fold higher kidney ANG II concentrations as compared with untreated non-induced rats (Figure 4C). 20-HETE antagonist did not alter ANG II levels in non-induced rats, AT_1_ receptor antagonist significantly decreased kidney ANG II in non-induced as well as in I3C-induced rats.

**Figure 5:**
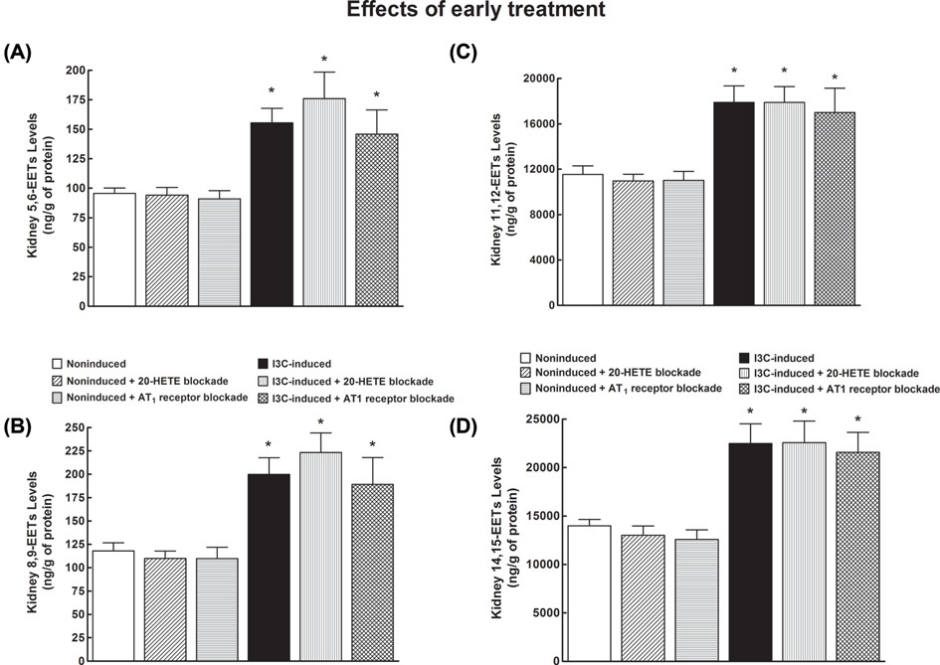
EETs levels in the kidney (**A**) Kidney 5,6-EETs, (**B**) kidney 8,9-EETs, (**C**) kidney 11,12-EETs, and (**D**) kidney 14,15-EETs concentrations in I3C-induced and non-induced Cyp1a1-Ren-2 transgenic rats, and effects of 20-HETE antagonist and AT_1_ receptor antagonist treatment. *n*=6–8 per group. *P<0.05 compared with untreated non-induced rats.

As shown in Figure 5, kidney 5,6-EET; 8,9-EET; 11,12-EET; and 14,15-EET were significantly higher in untreated I3C-induced as compared with untreated non-induced rats, and the treatment with 20-HETE antagonist or AT_1_ receptor antagonist did not change their intrarenal concentrations, similarly in non-induced or I3C-induced rats. A comparison of absolute intrarenal concentration values for individual EETs confirms that in the kidney tissue 11,12-EET and 14,15-EET are the prevalent epoxy fatty acids of the CYP-dependent epoxygenase pathway [45,48,50]. Moreover, since the ratio of EETs/DHETEs is lower in I3C-induced rats (Figure 4B), it is clear that this is not the consequence of compromised endogenous EETs formation, which is actually higher, but the result of increased conversion of EETs into DHETEs.

### Series 2: ‘Late treatment’ protocol

**Figure 6:**
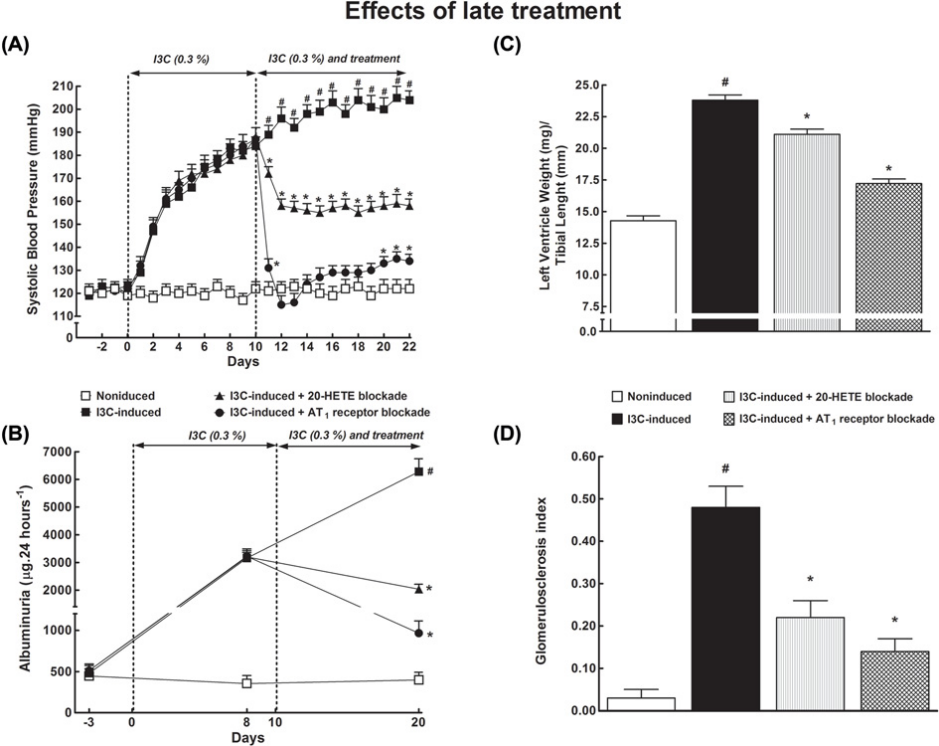
Comparison of 20-HETE receptor antagonist and ARB - late treatment Time course of SBP (**A**) and albuminuria (**B**), and the data for left ventricle weights normalized by tibial length (**C**) and glomerulosclerosis index (**D**) in I3C-induced and non-induced Cyp1a1-Ren-2 transgenic rats, and effects of 20-HETE antagonist or AT_1_ receptor antagonist treatment started during established hypertension (‘late treatment protocol’). *P<0.05 compared with basal values (or compared with non-induced rats in the case of left ventricle weights and glomerulosclerosis index). *n*=6–8 per group. ^#^*P*<0.05 compared with * marked values at the same time point (or compared with all other groups of rats in the case of left ventricle weights and glomerulosclerosis index).

Figure 6A shows that I3C induction for 10 days resulted in severe hypertension (SBP: 188 ± 4 mmHg). The treatment with 20-HETE receptor antagonist for 12 days resulted in a significant decrease in the pressure: at the end of experiment (day 22), it was substantially lower than in untreated I3C-induced rats (158 ± 2 compared with 204 ± 3 mmHg, *P*<0.05). The treatment with AT_1_ receptor antagonist brought SBP down to the normotensive range and the pressure remained low until the end of the experiment.

Figure 6B shows that in untreated I3C-induced rats, albuminuria progressively increased more than 12-fold as compared with basal values, and the treatment with 20-HETE antagonist or AT_1_ receptor antagonist substantially decreased it. However, the albuminuria still remained significantly higher than in untreated non-induced rats.

Figures 6C,D show that the treatment with 20-HETE antagonist or AT_1_ receptor antagonist attenuated cardiac hypertrophy and renal glomerular damage in I3C-induced rats.

**Figure 7:**
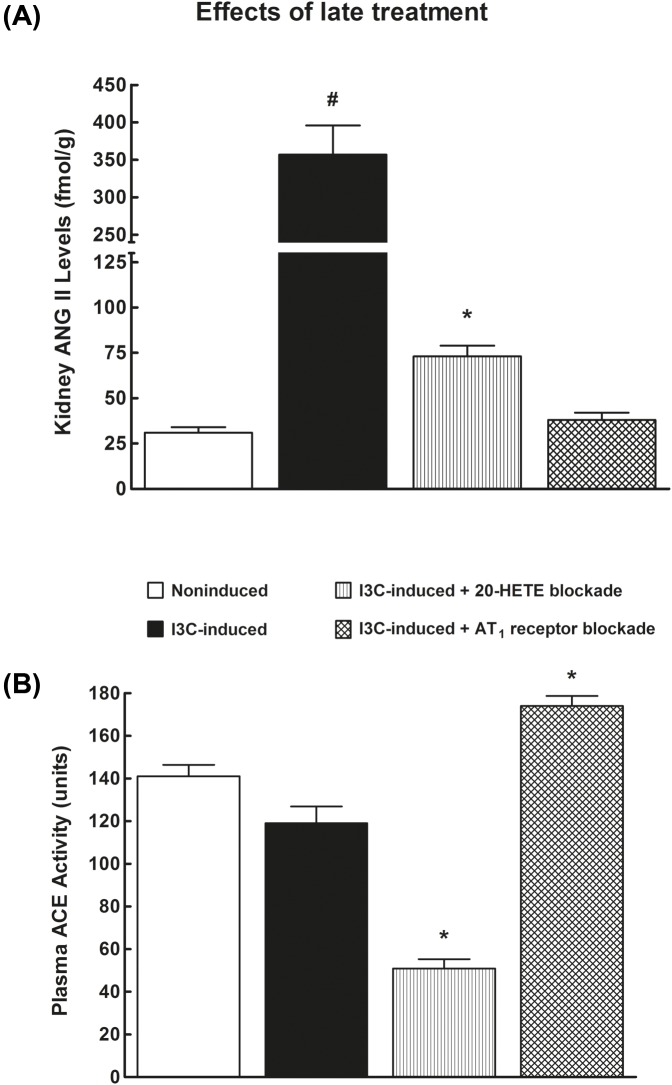
Comparison of 20-HETE receptor antagonist and ARB - late treatment Kidney ANG II levels (**A**) and plasma ACE activity (**B**) in I3C-induced and non-induced Cyp1a1-Ren-2 transgenic rats, and effects of 20-HETE antagonist or AT_1_ receptor antagonist treatment started during established hypertension (‘late treatment protocol’). *n*=6–8 per group. *P<0.05 compared with untreated non-induced rats. ^#^*P*<0.05 compared with all other values.

As shown in Figure 7A, in I3C-induced rats kidney ANG II level was 14-fold higher than in their non-induced counterparts. The treatment with 20-HETE antagonist significantly decreased both circulating (51 ± 7 compared with 152 ± 11 fmol.ml^−1^) and intrarenal ANG II (73 ± 6 compared with 357 ± 39 fmol.g^−1^) in I3C-induced rats in comparison with untreated I3C-induced rats. AT_1_ receptor antagonist also substantially decreased renal (Figure 7A) but not circulating ANG II levels. Plasma ACE activity did not significantly differ between non-induced and untreated I3C-induced rats (Figure 7B). Notably, the treatment with 20-HETE antagonist substantially decreased plasma ACE whereas the treatment with AT1 receptor antagonist induced its modest increase as compared with the values for non-induced or untreated 13C-induced rats.

### Series 3: ‘Renal hemodynamics and vascular responsiveness in late treatment’ protocol

Baseline renal hemodynamic parameters and maximal RBF response to ANG II (10 ng), ACh (50 ng), and l-NAME (50 μg.min^−1^.kg^−1^) are shown in Table 1. The responses to high-dose boluses of ANG II (20 ng) and ACh (100 ng) were greater than the respective responses to low doses. As we observed the same patterns of the renal vascular responses to low- and high-dose boluses, we present only the RBF responses to low-dose boluses for better lucidity of the table.

**Table 1:** Baseline renal hemodynamic parameters and maximal acute vascular responses to ANG II (10 ng), ACh (50 ng), and l-NAME

	*n*	RBF (ml.min^−1^.g^−1^)	RVR (mmHg/ml.min^−1^.g^−1^)	GFR (ml.min^−1^.g^−1^)	ANG II (% change in RBF)	ACh (% change in RBF)	l-NAME (% change in RBF)
Non-induced/untreated	8	9.65 ± 0.42	13 ± 1	0.92 ± 0.07	53 ± 6	16 ± 2	−63 ± 3
I3C-induced/untreated	9	5.94 ± 0.89^1^	29 ± 3^1^	0.75 ± 0.08	−8 ± 1^1^	9 ± 2^1^	−73 ± 3
I3C-induced/20-HETE antagonist	8	9.98 ± 0.77^2^	16 ± 2^2^	0.83 ± 0.06	−45 ± 4^2^	18 ± 2^2^	−64 ± 2
I3C-induced/AT_1_ receptor blocker	8	9.59 ± 0.75^2^	14 ± 1^2^	0.94 ± 0.07	−6 ± 2^1^,^3^	17 ± 3^2^	−60 ± 3^2^

Values are mean ± S.E.M. Abbreviation: I3C-induced, Cyp1a1-Ren-2 transgenic rats fed diet containing 0.3% I3C.

1 *P*<0.05 compared with non-induced rats.

2 *P*<0.05 compared with untreated I3C-induced rats.

3 *P*<0.05 compared with untreated I3C-induced rats/20-HETE antagonist.

## Discussion

The first important finding of the present study is that 20-HETE antagonist attenuated the development of malignant hypertension in Cyp1a1-Ren-2 transgenic rats. Beside the reduction in SBP, albuminuria, the degree of renal glomerular damage, and cardiac hypertrophy were also diminished. Even more important is our second finding that the treatment with 20-HETE antagonist-reduced SBP, the degree of renal glomerular damage, and cardiac hypertrophy and restored renal hemodynamic parameters and renal vascular responsiveness in the rats with already established malignant hypertension. Taken together, the findings suggest that alterations in 20-HETE production and/or action critically contribute to the development of hypertension and hypertensive end-organ damage in this form of ANG II-dependent malignant hypertension. The crucial issue is what are the exact mechanism(s) underlying the beneficial effects of 20-HETE blockade.

Since the studies of the past two decades have shown that 20-HETE has both pro- and antihypertensive properties, the consequences of 20-HETE blockade on BP are difficult to predict. It is generally accepted that prohypertensive actions of 20-HETE are related to its vascular effects [16–20]. This agent promotes vasoconstriction via mechanisms involving inhibition of large-conductance, calcium-activated potassium (BK_Ca_) channels [51], increasing calcium channel conductance due to depolarization of the arteriolar vascular smooth muscle [52] and activation of Rho-kinase which causes sensitization of the contractile apparatus to calcium [53]. In addition, it has been shown that 20-HETE augments vascular reactivity to various vasoconstrictor agents [54,55]. Moreover, it plays an important role in the mediation of acute and chronic pressor effects of ANG II [22,25,46,56,57]. Furthermore, several studies suggested positive feedback interactions between vascular CYP450/20-HETE system and the RAS [20–24] and more recently it was proposed that 20-HETE might serve as an upstream effector molecule for activation of the RAS, and the first specific receptor for 20-HETE was identified [28,29]. Our results indicate that the treatment with 20-HETE receptor antagonist displayed predominantly vascular effects in the kidney to restore altered renal vascular resistance, RBF, and renal vascular responsiveness in hypertensive Cyp1a1-Ren-2 transgenic rats.

Dissimilarly, antihypertensive actions of 20-HETE are related to its action on the renal tubule [17–19,57]. It inhibits sodium reabsorption in its proximal segment by blocking sodium-potassium-ATPase (Na^+^-K^+^-ATPase), and in the thick ascending limb of the loop of Henle (TALH) via inhibiting Na-K-2Cl co-transporter or, indirectly, by inhibiting Na^+^-K^+^-ATPase and a local rise in intracellular concentration of Na^+^ [58,59]. Whatever the actual mechanism, 20-HETE inhibits sodium transport and, thereby, reduces extracellular fluid volume, which should oppose rather than promote hypertension [19,60]. This notion is strengthened by findings that chronic blockade of renal formation of 20-HETE is associated with the development of salt-sensitive hypertension in otherwise salt-resistant rats [61], and also by the demonstration that genetic or pharmacological enhancement of renal formation of 20-HETE improves the efficiency of the pressure-natriuresis mechanism and attenuates the development of hypertension in various experimental models [62].

In view of the uncertainty regarding the role of intrarenal 20-HETE in hypertension (net prohypertensive or antihypertensive?), its role in the pathophysiology of ANG II-malignant hypertension has been so far disregarded.

Our results indicating augmented renal and peripheral vascular resistance in Cyp1a1-Ren-2 transgenic rats might suggest that prohypertensive actions of 20-HETE and its interaction with RAS importantly contribute to the pathophysiology of malignant hypertension in this model [33,43,44]. However, this interpretation seems difficult to reconcile with our present and recent findings that after induction of *renin* gene intrarenal concentrations of 20-HETE are actually decreased in Cyp1a1-Ren-2 transgenic rats. Similar to our previous study [40], we observed that lower renal levels of 20-HETE in our model of malignant hypertension does not depend on SBP or ANG II levels. Both 20-HETE receptor antagonist and AT1 receptor inhibitor did not affected 20-HETE levels in the kidney in Cyp1a1-Ren-2 transgenic rats. This may indicate that 20-HETE formation is regulated by other pathways, most likely by direct alteration of CYP450 enzymes, at least in this model of malignant hypertension. It is noteworthy that ANG II-induced 20-HETE generation is also diminished in the kidney failure model [63]. Therefore it remains essential to understand the link between renal 20-HETE, renal ANG II concentrations in terms of BP regulation.

It is also important to recognize that intrarenal concentrations (measured in the cortex homogenate) do not reflect the rate of 20-HETE formation and availability in renal resistance vessels but rather its production and accumulation in the renal tubules [57]. Therefore, the measured intrarenal deficiency of 20-HETE (reflecting mostly decreased intratubular content) and the consequent elimination of 20-HETE-mediated inhibition of tubular reabsorption may further promote the development of ANG II-dependent malignant hypertension in this model, which would agree with our recent report on the beneficial effects of fenofibrate [40]. This interpretation is also supported by the evidence that fenofibrate increases renal formation of 20-HETE. Since blood vessels do not express the peroxisome proliferator-activated receptor α, fenofibrate does not increase vascular production of 20-HETE [62,64], hence the treatment decreased BP in various experimental models even though vascular 20-HETE concentration remained elevated [17–20,62]. Furthermore, we reported repeatedly that the development of ANG II-dependent malignant hypertension in Cyp1a1-Ren-2 transgenic rats is accompanied by decreased intrarenal availability of EETs [34,40,43–45]. These agents importantly diminish the tone of peripheral resistance vessels and inhibit renal tubular sodium transport and, therefore, are implicated in long-term BP control [19,60,65]. We provided evidence that the intrarenal EETs deficiency significantly contributes to the impairment of renal function and the development and maintenance of malignant hypertension in I3C-induced Cyp1a1-Ren-2 transgenic rats [43–45].

Taken together, our previous and present results support the thesis that prohypertensive actions of 20-HETE in the vasculature combined with deficiency of antihypertensive effects of 20-HETE and EETs at the tubular level as well as diminished renal vasodilatory action of EETs act in concert to promote the development of ANG II-dependent malignant hypertension, at least its form found in Cyp1a1-Ren-2 transgenic rats.

Notwithstanding the above compelling evidence, one must, however, be aware that, as reported by us and others malignant hypertension in the Cyp1a1-Ren-2 transgenic rats is characterized by a distinct intrarenal augmentation of the hypertensogenic axis of the RAS, as reflected by a marked increase in kidney ANG II concentration [31–36]. Most probably, this feature is of crucial pathophysiological importance whereas the aforementioned alterations in CYP450-derived metabolites of arachidonic acid represent only permissive contributing factor in this model. Nevertheless, our present findings further support the view that altered intrarenal interactions of the RAS with other vasoactive systems play an important role in the pathophysiology of malignant hypertension.

Of considerable interest is also our finding that chronic treatment with 20-HETE antagonist suppressed kidney ANG II levels. Assuming the critical role of the enhanced RAS activity in the development and maintenance of hypertension in Cyp1a1-Ren-2 transgenic rats [30–32,34–36,39,40], it seems plausible that the suppression of augmented intrarenal ANG II levels, both within the early and the late treatment protocols, is the main factor in antihypertensive actions of 20-HETE antagonist in our study. This observation supports the notion that there is a close interaction between 20-HETE and RAS activity. Besides evidence of positive feedback interactions, 20-HETE seems to contribute directly to ANG II vasoconstriction, vascular remodeling, and inflammatory processes [19,27,57,60]. Therefore the organoprotection effect of the 20-HETE antagonist treatment observed also in the present study might be predominantly attributed to lowering of ANG II concentration accompanied by significant reduction in SBP in this model. It has to be noted that induction of hypertension in Cyp1a1-Ren-2 transgenic rats is fully ANG II-dependent with exaggerated circulating as well as renal ANG II levels. 20-HETE receptor antagonist markedly affected both these levels which led to SBP lowering. In contrast, AT_1_ receptor blocker lowered renal but not circulating ANG II level and SBP was almost normalized in I3C-induced rats.

The fact that 20-HETE, as a potent inducer of ACE, modulates the RAS activity and thus contributes in the pathophysiology of cardiovascular diseases is now well recognized [20,23,27,28]. We showed here that 20-HETE receptor antagonist markedly suppressed ACE activity in I3C-induced rats. This is very important because intrarenal ANG II content in ANG II-dependent models of hypertension results from both AT_1_ receptor-mediated uptake of circulating ANG II and *de novo* intrarenal ANG II formation [66]; this is also the case with the Cyp1a1-Ren-2 transgenic rats after induction of malignant hypertension [31,35,36]. Since Gonzalez-Villalobos et al. [67] have demonstrated that ACE-mediated ANG II formation is important for the progressive increase in intrarenal ANG II levels and the development of hypertension, one can assume that 20-HETE receptor antagonist diminished the ACE activity leading to the reduction in ACE-mediated ANG II generation and the suppression of the intrarenal ANG II levels. Moreover, 20-HETE antagonist completely restored the vascular responsiveness to ANG II in our model that indicates an important role of 20-HETE in the regulation of RAS activity. Therefore we suggest that chronic treatment with 20-HETE receptor antagonist directly alters the circulating and intrarenal RAS, which resulted in lowering plasma or renal ANG II levels via regulation of ACE activity, modulates sensitivity of AT1 receptors, and restores renal hemodynamics in hypertensive Cyp1a1-Ren-2 transgenic rats. This might be the main mechanism responsible for BP lowering, both in the early an in late treatment protocol, at least in the present model of ANG II-dependent malignant hypertension.

## Clinical perspectives

- There is a great need for new therapies of malignant hypertension, but this requires more detailed understanding of the pathophysiology of this form of the disease.
- The present study shows that the 20-HETE receptor antagonist treatment of rats with ANG II-dependent malignant hypertension attenuated the development and largely reversed the established disease, likely via suppression of intrarenal ANG II levels. Thus, interaction between 20-HETE and the RAS plays here an important pathophysiological role.
- Our findings provide experimental basis for new pharmacological approaches or tools for the treatment of malignant form of hypertension.

## Abbreviations

AAA: 20-HETE receptor antagonist
ACE: angiotensin-converting enzyme
ACh: acetylcholine
ANG II: angiotensin II
AT_1_: ANG II type 1
BP: blood pressure
BW: body weight
CYP450: cytochrome P450
DHETE: dihydroxyeicosatrienoic acid
EET: epoxyeicosatrienoic acid
20-HETE: 20-hydroxyeicosatetraenoic acid
I3C: indole-3-carbinol
l-NAME: N^ω^-nitro-l-arginine methyl ester
RAS: renin–angiotensin system
RBF: renal blood flow
SBP: systolic BP

## References

[1] Poulter N.R., Prabhakaran D. and Culfield M. (2015) Hypertension. Lancet 386, 801–812 10.1016/S0140-6736(14)61468-9 25832858

[2] Rossignol P., Massy Z.A., Azizi M., Bakris G., Ritz E. and Covic A. (2015) The double challenge of resistant hypertension and chronic kidney disease.. Lancet 386, 1588–1598 10.1016/S0140-6736(15)00418-3 26530623

[3] Lip G.Y., Beevers M. and Beevers D.G. (1995) Complications and survival of 315 patients with malignant-phase hypertension. J. Hypertens. 13, 915–924 10.1097/00004872-199508000-00013 8557970

[4] Guerin C., Gonthier R. and Berthoux F.C. (1988) Long-term prognosis in malignant or accelerated hypertension. Nephrol. Dial. Transplant. 3, 33–37 3132637

[5] Lane D.A., Lip G.Y.H. and Beevers D.G. (2009) Improving survival of malignant hypertension patients over 40 years. Am. J. Hypertens. 22, 1199–1204 10.1038/ajh.2009.153 19696746

[6] Januszewicz A., Guzik T., Prejbisz A., Mikolajczyk T., Osmenda G. and Januszewicz W. (2016) Malignant hypertension: new aspects of an old clinical entity. Pol. Arch. Med. Wewn. 126, 86–93 26658350

[7] Cremer A., Amraoui F., Lip G.Y., Morales E., Rubin S., Sequra J. (2016) From malignant hypertension to hypertension-MOD: a modern definition for old but still dangerous emergency. J. Hum. Hypertens. 30, 463–466 10.1038/jhh.2015.112 26582411

[8] Mancia G., Fagard R., Narkiewicz K., Redán J., Zanchetti A., Böhm M. (2013) Practice guidelines for the management of arterial hypertension of the European Society of Hypertension (ESH) and European Society of Cardiology (ESC): ESH/ESC Task Force for the Management of Arterial Hypertension. J. Hypertens. 31, 1925–1938 10.1097/HJH.0b013e328364ca4c 24107724

[9] Laragh J.H. and Sealey J.E. (1990) Renin system understanding for analysis and treatment of hypertensive patients: a means to quantify the vasoconstrictor elements, diagnose curable renal and adrenal causes, assess risk of cardiovascular morbidity, and find the best-fit drug regimen. In Hypertension: Pathophysiology, Diagnosis and Management (Laragh J.H. and Brenner B.M., eds), pp. 1813–1836, Raven Press Publishers, New York, N.Y.

[10] Laragh J.H. (2001) Laragh’s lessons in pathophysiology and clinical pearls for treating hypertension. Am. J. Hypertens. 14, 186–194 10.1016/S0895-7061(00)01317-0 11243312

[11] Kincaid-Smith P. (1991) Malignant hypertension. J. Hypertens. 9, 893–899 10.1097/00004872-199110000-00002 1658131

[12] Fleming S. (2000) Malignant hypertension – the role of the paracrine renin-angiotensin system. J. Pathol. 192, 135–139 10.1002/1096-9896(2000)9999:9999%3c::AID-PATH674%3e3.0.CO;2-Q 11004688

[13] Collidge T.A., Lammie G.A., Fleming S. and Mullins J.J. (2004) The role of the renin-angiotensin system in malignant vascular injury affecting the systemic and cerebral circulations. Prog. Biophys. Mol. Biol. 84, 301–319 10.1016/j.pbiomolbio.2003.11.003 14769441

[14] Liu X., Bellamy C.O., Bailey M.A., Mullins L.J., Dunbar D.R. and Kenyon C.J. (2009) Angiotensin-converting enzyme is a modifier of hypertensive end organ damage. J. Biol. Chem. 284, 15564–15572 10.1074/jbc.M806584200 19307186 PMC2708853

[15] Hall J.E., Granger J.P. and Hall M.E. (2013) Physiology and pathophysiology of hypertension. In Seldin and Giebisch’s The Kidney Physiology and Pathophysiology, 5th edn (Albpern R.J., Caplan M.J. and Moe O.W., eds), pp. 1319–1352, Academic Press

[16] Capdevila J.H., Wang W. and Falck J.R. (2015) Arachidonic acid monooxygenase: genetic and biochemical approaches to physiological/pathophysiological relevance. Prostaglandins Other Lipid Mediat. 120, 40–49 10.1016/j.prostaglandins.2015.05.004 25986599 PMC4575609

[17] Fan F., Ge Y., Lv M., Elliot M.R., Muroya Y., Hirata T. (2016) Molecular mechanisms and cell signaling of 20-hydroxyeicosatetraenoic acid in vascular pathophysiology. Front. Biosci. 21, 1247–1463 10.2741/4465 PMC5064940 27100515

[18] Waldman M., Peterson S.J., Arad M. and Hochhauser E. (2016) The role of 20-HETE in cardiovascular diseases and its risk factors. Prostaglandins Other Lipid Mediat. 125, 108–117 10.1016/j.prostaglandins.2016.05.007 27287720

[19] Imig J.D. (2016) Epoxyeicosatrienoic acids and 20-hydroxyeicosatetraenoic acid on endothelial and vascular function. Adv. Pharmocol. 77, 105–141 10.1016/bs.apha.2016.04.003 PMC5510644 27451096

[20] Garcia V. and Schwartzman M.L. (2017) Recent developments on the vascular effects of 20-hydroxyeicosatetraenoic acid. Curr. Opin. Nephrol. Hypertens. 26, 74–82 27906746 10.1097/MNH.0000000000000302

[21] Croft K.D., McGiff J.C., Sanchez-Mendoza A. and Carroll M.A. (2000) Angiotensin II releases 20-HETE from rat renal microvessels. Am. J. Physiol. 279, F544–F551 10.1152/ajprenal.2000.279.3.F544 10966934

[22] Joly E., Seqqat R., Flamion F., Caron N., Michel A., Imig J.D. (2006) Increased renal vascular reactivity to ANG II after unilateral nephrectomy in the rat involves 20-HETE. Am. J. Physiol. 291, R977–R986 10.1152/ajpregu.00401.2005 16675634

[23] Sodhi K., Wu C.C., Cheng J., Gotlinger K., Inoue K., Goli M. (2010) CYP4A2-induced hypertension is 20-hydroxyeicosatetraenoic acid- and angiotensin II-dependent. Hypertension 56, 871–878 10.1161/HYPERTENSIONAHA.110.154559 20837888 PMC2995375

[24] Cheng J., Garcia V., Ding Y., Wu C.C., Thakra K., Falck J.R. (2012) Induction of angiotensin-convertng enzyme and activation of the renin-angiotensin system contribute to 20-hydroxyeicosatetraenoic acid-mediated endothelial dysfunction. Arterioscler. Thromb. Vasc. Biol. 32, 1917–1924 10.1161/ATVBAHA.112.248344 22723444 PMC3418056

[25] Fan F., Sun C.W., Maier K.G., Williams J.M., Pabbidi M.R., Didion S.P. (2013) 20-hydroxyeicosatetraenoic acid contributes to the inhibition of K^+^ channel activity and vasoconstrictor responses to angiotensin II in rat renal microvessles. PLoS ONE 8, e82482 10.1371/journal.pone.0082482 24324797 PMC3853207

[26] Garcia V., Joseph G., Shkolnik B., Ding Y., Zhang F.F., Gotlinger K. (2015) Angiotensin II receptor blockade or deletion of vascular endothelial ACE does not prevent vascular dysfunction and remodeling in 20-HETE-dependent hypertension. Am. J. Physiol. 309, R71–R78 10.1152/ajpregu.00039.2015 PMC4491537 25924878

[27] Savas U., Wei S., Hsu M.H., Falck J.R., Guengerich F.P., Capdevilla J.H. (2016) 20-hydroxyeicosatetraenoic acid (HETE)-dependent hypertension in human cytochrome P450 (CYP)4A11 transgenic mice: normalization of blood pressure by sodium restriction, hydrochlothiazide, or blockade of the type 1 angiotensin II receptor. J. Biol. Chem. 291, 16904–16919 10.1074/jbc.M116.732297 27298316 PMC4974402

[28] Garcia V., Shkolik B., Milhau L., Falck J.R. and Schwartzman M.L. (2016) 20-HETE activates the transcription of angiotensin-converting enzyme via nuclear factor-kB translocation and promoter binding. J. Pharmacol. Exp. Ther. 356, 525–533 10.1124/jpet.115.229377 26699146 PMC4767392

[29] Garcia V., Gilani A., Shkolnik B., Pandey V., Zhang F.F., Dakarapu R. (2017) 20-HETE signals through G-protein-coupled receptor GPR75 (G_q_) to affect vascular function and trigger hypertension. Circ. Res. 120, 1776–1788 10.1161/CIRCRESAHA.116.310525 28325781 PMC5446268

[30] Kantachuvesiri S., Fleming S., Peters J., Peters B., Brooker G., Lammie A.G. (2001) Controlled hypertension, a transgenic toggle switch reveals differential mechanisms underlying vascular disease. J. Biol. Chem. 276, 36727–36733 10.1074/jbc.M103296200 11448960

[31] Vaňourková Z., Kramer H.J., Husková Z., Vaněčková I., Opočenský M., Čertíková Chábová V. (2006) AT_1_ receptor blockade is superior to conventional triple therapy in protecting against end-organ damage in Cyp1a1-Ren-2 transgenic rats with inducible hypertension. J. Hypertens. 24, 2465–2472 10.1097/01.hjh.0000251909.00923.22 17082731

[32] Husková Z., Vaňourková Z., Erbanová M., Thumová M., Opočenský M., Mullins J.J. (2010) Inappropriately high circulating and intrarenal angiotensin II levels during dietary salt loading exacerbate hypertension in Cyp1a1-Ren-2 transgenic rats. J. Hypertens. 28, 495–509 10.1097/HJH.0b013e3283345d69 19927008

[33] Erbanová M., Thumová M., Husková Z., Vaněčková I., Vaňourková Z., Mullins J.J. (2009) Impairment of the autoregulation of renal hemodynamics and of the pressure-natriuresis relationship precedes the development of hypertension in Cyp1a1-Ren-2 transgenic rats. J. Hypertens. 27, 575–586 10.1097/HJH.0b013e32831cbd5a 19330918

[34] Sporková A., Jíchová Š, Husková Z., Kopkan L., Nishiyama A., Hwang S.H. (2014) Different mechanisms of acute versus long-term antihypertensive effects of soluble epoxide hydrolase inhibition: studies in Cyp1a1-Ren-2 transgenic rats. Clin. Exp. Pharmacol. Physiol. 41, 1003–1013, 10.1111/1440-1681.12310 25224811 PMC4347520

[35] Mitchell K.D., Bagatell S.J., Miller C.S., Mouton C.R., Seth D.M. and Mullins J.J. (2006) Genetic clamping of renin gene expression induces hypertension and elevation of intrarenal angiotensin II levels of graded severity in Cyp1a1-Ren2 transgenic rats. J. Renin Angiotensin Aldosterone Syst. 7, 74–86 17083061 10.3317/jraas.2006.013

[36] Williams D.E., Prieto M.C., Mullins J.J., Navar L.G. and Mitchell K.D. (2010) AT_1_ receptor blockade prevents the increase in blood pressure and the augmentation of intrarenal ANG II levels in hypertensive Cyp1a1-Ren-2 transgenic rats fed a high salt diet. Am. J. Med. Sci. 339, 356–361 10.1097/MAJ.0b013e3181d2b0a8 20224314 PMC2880537

[37] Fleming I. (2014) The pharmacology of the cytochrome P450 epoxygenase/soluble epoxide hydrolase axis in the vasculature and cardiovascular disease. Pharmacol. Rev. 66, 1106–1140 10.1124/pr.113.007781 25244930

[38] Whitworth C.E., Fleming S., Kotelevtsev Y., Manson L., Brooker G.A., Cumming A.D. (1995) A genetic model of malignant phase hypertension in rats. Kidney Int. 47, 529–535 10.1038/ki.1995.66 7723238

[39] Husková Z., Kopkan L., Červenková L., Doleželová Š, Vaňourková Z., Škaroupková P. (2016) Intrarenal alterations of the angiotensin-converting enzyme type 2/angiotensin 1-7 complex of the renin-angiotensin system do not alter the course of malignant hypertension in Cyp1a1-Ren-2 transgenic rats. Clin. Exp. Pharmacol. Physiol. 43, 438–449 10.1111/1440-1681.12553 26833491

[40] Jíchová Š, Doleželová Š, Kopkan L., Kompanowska-Jezierska E., Sadowski J. and Červenka L. (2016) Fenofibrate attenuates malignant hypertension by suppression of the renin-angiotensin system: a study in Cyp1a1-Ren-2 transgenic rats. Am. J. Med. Sci. 352, 618–630 10.1016/j.amjms.2016.09.008 27916218

[41] Gangadhariah M., Luther J.M., Garcia V., Paueksakon P., Zhang M.Z., Hayward S.W. (2015) Hypertension is a major contributor to 20-hydroxyeicosatetraenoic acid-mediated kidney injury in diabetic nephropathy. J. Am. Soc. Nephrol. 26, 597–610 10.1681/ASN.2013090980 25071086 PMC4341468

[42] Kurtz T.W., Griffin K.A., Bidani A.K., Davisson R.L. and Hall J.E. (2005) Recommendations for blood pressure measurements in humans and experimental animals. Part 2: Blood pressure measurements in experimental animals. Hypertension 45, 299–310 10.1161/01.HYP.0000150857.39919.cb 15611363

[43] Honetschlägerová Z., Husková Z., Vaňourková Z., Sporková A., Kramer H.J., Hwang S.H. (2011) Renal mechanisms contributing to the antihypertensive action of soluble epoxide hydrolase inhibition in Ren-2 transgenic rats with inducible hypertension. J. Physiol. 589, 207–219 10.1113/jphysiol.2010.199505 21078594 PMC3039270

[44] Honetschlägerová Z., Sporková A., Kopkan L., Husková Z., Hwang S.H., Hammock B.D. (2011) Inhibition of soluble epoxide hydrolase improves the impaired pressure-natriuresis relationship and attenuates the development of hypertension and hypertension-associated end-organ damage in Cyp1a1-Ren-2 transgenic rats. J. Hypertens. 29, 1590–1601 10.1097/HJH.0b013e328349062f 21720266 PMC3777565

[45] Jíchová Š, Kopkan L., Husková Z., Doleželová Š, Neckář J., Kujal P. (2016) Epoxyeicosatrienoic acid analog attenuates the development of malignant hypertension, but does not reverse it once established: a study in Cyp1a1-Ren-2 transgenic rats. J. Hypertens. 34, 2008–2025 10.1097/HJH.0000000000001029 27428043 PMC5510029

[46] Čertíková Chábová V., Walkowska A., Kompanowska-Jezierska E., Sadowski J., Kujal P., Vernerová Z. (2010) Combined inhibition of 20-hydroxyeicosateraenoic acid formation and of epoxyeicosatrienoic acids degradation attenuates hypertension and hypertension-induced end-organ damage in Ren-2 transgenic rats. Clin. Sci. 118, 617–632 10.1042/CS20090459 20050826 PMC2854172

[47] Yin FCP, Spurgeon H.A., Rakusan K., Weisfeldt M.L. and Lakatta E.G. (1982) Use of tibial length to quantify cardiac hypertrophy: application in the aging rat. Am. J. Physiol. 243, H941–H947 6216817 10.1152/ajpheart.1982.243.6.H941

[48] Husková Z., Kramer H.J., Vaňourková Z. and Červenka L. (2006) Effects of changes in sodium balance on plasma and kidney angiotensin II levels in anesthetized and conscious Ren-2 transgenic rats. J. Hypertens. 24, 517–527 10.1097/01.hjh.0000209988.51606.c7 16467655

[49] Husková Z., Kramer H.J., Thumová M., Vaňourková Z., Burgerová M., Teplan V. (2006) Effects of anesthesia on plasma and kidney ANG II levels in normotensive and ANG II-dependent hypertensive rats. Kidney Blood Pres. Res. 29, 74–83 10.1159/000092981 16651849

[50] Sporková A., Čertíková Chábová V., Doleželová Š, Jíchová Š, Kopkan L., Vaňourková Z. (2017) Fenofibrate attenuates hypertension in Goldblatt hypertensive rats: role of 20-hydroxyeicosatetraenoic acid in the nonclipped kidney. Am. J. Med. Sci. 353, 568–579 10.1016/j.amjms.2017.04.009 28641720

[51] Zou A.P., Fleming J.T., Falck J.R., Jacobs E.R., Gebremedhin D., Harder D.R. (1996) 20-HETE is an endogenous inhibitor of the large-conductance Ca^2+^-activated K^+^ channel in renal arterioles. Am. J. Physiol. 270, R228–R237 8769806 10.1152/ajpregu.1996.270.1.R228

[52] Randriamboavonjy V., Kiss L., Falck J.R., Busse R. and Fleming I. (2005) The synthesis of 20-HETE in small porcine coronary arteries antagonizes EDHF-mediated relaxation. Cardiovas. Res. 65, 487–494 10.1016/j.cardiores.2004.10.029 15639488

[53] Randriamboavonjy V., Busse R. and Fleming I. (2003) 20-HETE-induced contraction of small coronary arteries depends on the activation of Rho-kinase. Hypertension 41, 801–806 10.1161/01.HYP.0000047240.33861.6B 12623999

[54] Kaide J., Wang M.H., Wang J.S., Zhang F., Gopal V.R., Falck J.R. (2003) Transfection of CYP4A1 cDNA increases vascular reactivity in renal interlobar arteries. Am. J. Physiol. 284, F51–F56 10.1152/ajprenal.00249.2002 12388396

[55] Imig J.D., Pham B.T., LeBlanc E.A., Reddy K.M., Falck J.R. and Inscho E.W. (2000) Cytochrome P450 and cyclooxygenase metabolites contribute to the endothelin-1 afferent arteriolar vasoconstriction and calcium response. Hypertension 35, 307–312 10.1161/01.HYP.35.1.307 10642316

[56] Alonso-Galicia M., Maier K.G., Greene A.S., Cowley A.W. and Roman R.J. (2002) Role of 20-hydroxyeicosatetraenoic acid in the renal and vasoconstrictor actions of angiotensin II. Am. J. Physiol. 283, R60–R68 10.1152/ajpregu.00664.2001 12069931

[57] Roman R.J. (2002) P-450 metabolites of arachidonic acid in the control of cardiovascular function. Physiol. Rev. 82, 131–185 10.1152/physrev.00021.2001 11773611

[58] Quigley R., Baum M., Reddy K.M., Griener J.C. and Falck J.R. (2000) Effects of 20-HETE and 19(S)-HETE on rabbit proximal straight tubule volume transport. Am. J. Physiol. 278, F949–F953 10.1152/ajprenal.2000.278.6.F949 PMC4124896 10836982

[59] Yu M., Lopez B., Dos Santos E.A., Falck J.R. and Roman R.J. (2007) Effects of 20-HETE on Na^+^ transport and Na^+^-K^+^-ATPase activity in the thick ascending loop of Henle. Am. J. Physiol. 292, R2400–R2405 10.1152/ajpregu.00791.2006 17303679

[60] Fan F. and Roman R.J. (2017) Effect of cytochrome P450 metabolites of arachidonic acid in Nephrology. J. Am. Soc. Nephrol. 28, 2845–2855 10.1681/ASN.2017030252 28701518 PMC5619976

[61] Hoagland K.M., Flasch A.K. and Roman R.J. (2003) Inhibitors of 20-HETE formation promote salt-sensitive hypertension in rats. Hypertension 42, 669–673 10.1161/01.HYP.0000084634.97353.1A 12874093

[62] Williams J.M., Murphy S., Burke M. and Roman R.J. (2010) 20-hydroxyeicosatetraenoic acid: a new target for the treatment of hypertension. J. Cardiovasc. Phamarcol. 56, 336–344 10.1097/FJC.0b013e3181f04b1c PMC2953733 20930591

[63] Bautista R., Sánchez A., Hernández J., Oyekan A. and Escalante B. (2001) Angiotensin II type AT(2) receptor mRNA expression and renal vasodilatation are increased in renal failure. Hypertension 38, 669–673 10.1161/hy09t1.096186 11566953

[64] Vera T., Taylor M., Bohman Q., Flasch A., Roman R.J. and Stec D.E. (2005) Fenofibrate prevents the development of angiotensin II-dependent hypertension in mice. Hypertension 45, 730–735 10.1161/01.HYP.0000153317.06072.2e 15699464

[65] Hye Khan M.A., Pavlov T.S., Christain S.V., Neckář J., Staruschenko A. and Gauthier K.M. (2014) Epoxyeicosatrienoic acid analogue lowers blood pressure through vasodilatation and sodium channel inhibition. Clin. Sci. 127, 463–474 10.1042/CS20130479 24707975 PMC4167712

[66] Navar L.G., Prieto M.C., Satou R. and Kobori H. (2011) Intrarenal angiotensin II and its contribution to the genesis of chronic hypertension. Curr. Opin. Pharmacol. 11, 180–186 10.1016/j.coph.2011.01.009 21339086 PMC3075356

[67] Gonzalez-Villalobos R.A., Janjoulia T., Fletcher N.K., Giani J.F., Nguyen M.T.X. and Riquier-Brison A.D. (2013) The absence of intrarenal ACE protects against hypertension. J. Clin. Invest. 123, 2011–2023 10.1172/JCI65460 23619363 PMC3638907

